# Supplementary material for: Simultaneous Production of Cellulose Nitrates and Bacterial Cellulose from Lignocellulose of Energy Crop
Source: Polymers (Basel). 2023 Dec 21;16(1):42. doi: 10.3390/polym16010042 (PMC10780700; doi:10.3390/polym16010042)
Supplement: Supplementary file 1 [file polymers-16-00042-s001.zip › polymers-2768095-supplementary.pdf]

Supplementary Materials

Simultaneous Production of Cellulose Nitrates and Bacterial Cellulose from Lignocellulose of Energy Crop

Ekaterina I. Kashcheyeva*, Anna A. Korchagina, Yulia A. Gismatulina, Evgenia K. Gladysheva*, Vera V. Budaeva, Gennady V. Sakovich

Bioconversion Laboratory, Institute for Problems of Chemical and Energetic Technologies, Siberian Branch of the Russian Academy of Sciences (IPCET SB RAS), 659322 Biysk, Russia

**Correspondence: makarova@ipcet.ru (E.I. Kashcheyeva), ORCID: 0000-0003-1593-7982; evg-gladysheva@yandex.ru (E.K. Gladysheva), ORCID: 0000-0002-6567-9662.*

Table of Contents

Experimental design in brief	S2
**Figure S1.** Experimental scheme	S3
**Figure S2.** SEM images of *Miscanthus* cellulose fibers with pores after enzymatic hydrolysis	S4-S5

The experimental design is graphically represented in brief in Figure S1 with the designated resultant samples, intermediates and target products. The names of the intermediates and target products are marked up with bold font herein.

Cellulose (**C_0 cellulose**) was isolated from the ground Miscanthus (**Miscanthus**) by the successive treatment of the feedstock with dilute solutions of nitric acid and sodium hydroxide at 90–95 °C. It was found at the first stage of the study on the enzymatic hydrolysis of Miscanthus cellulose that the enzymatic hydrolysis of the Miscanthus cellulose sample (**C_0 cellulose**) must be performed for 2, 8 and 24 h. Upon completion of the process, the resultant reaction mass was subjected to filtration followed by carefully washing the solid residue which was further dried. The solid cellulose residues from each of the experiments were designated as **C2**, **C8** and **C24**, respectively. These cellulose samples, **C2**, **C8** and **C24**, were further analyzed for cellulose and then nitrated. The liquid phases (enzymatic hydrolyzates) from each of the experiments were designated in a similar manner with the added word "**hydrolyzate**" as follows: **C2 hydrolyzate, C8 hydrolyzate** and **C24 hydrolyzate**. All the three hydrolyzates were then utilized to prepare nutrient media to subsequently synthesize bacterial cellulose (BC).

Four cellulose samples, including the initial one, namely **C_0, C2, C8** and **C24** c**ellulose,** were nitrated by the common sulfuric-nitric acid process by treating the starting cellulose samples with a commercial sulfuric-nitric acid mixture to consequently furnish the four **cellulose nitrate** (CN) samples: **CN_0**, **CN2**, **CN8** and **CN24**, respectively. All the four CN samples were analyzed and compared with each other.

The three enzymatic hydrolyzates—**C2, C8** and **C24 hydrolyzates**—were employed to prepare a nutrient medium followed by biosynthesis of BC samples. The BC samples were designated in the scheme below according to their enzymatic hydrolysis time as follows: **BC2**, **BC8** and **BC24**.

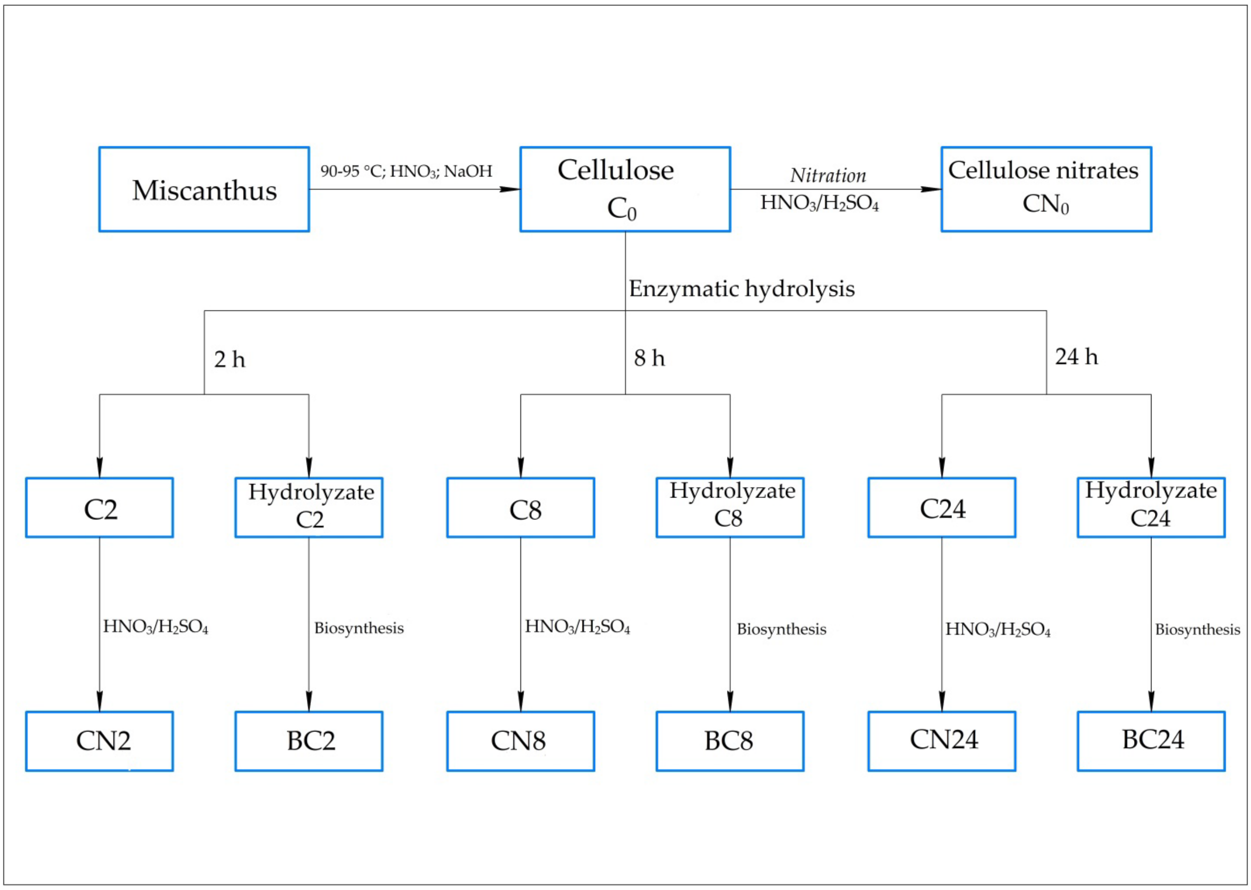

Figure S1. The graphical representation of the experimental scheme in brief

Figure S2 shows microphotographs (×200 and ×5,000 zoom) of *Miscanthus* cellulose fibers with pores after enzymatic hydrolysis.

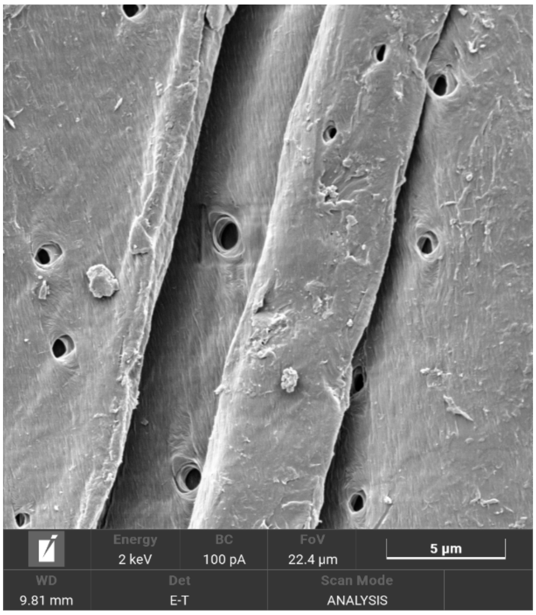

(a)

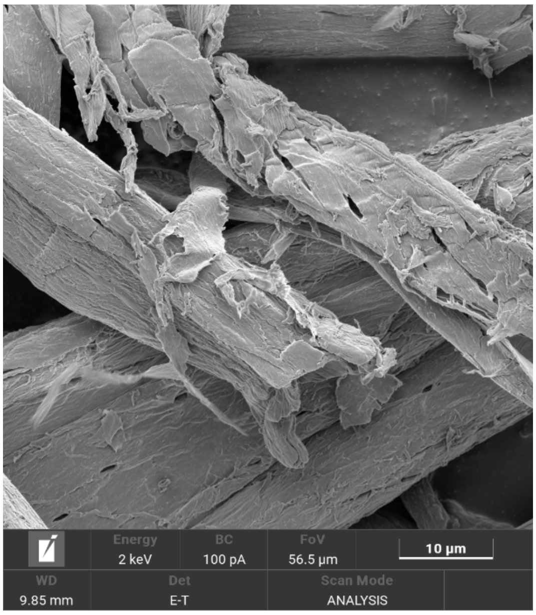

(b)

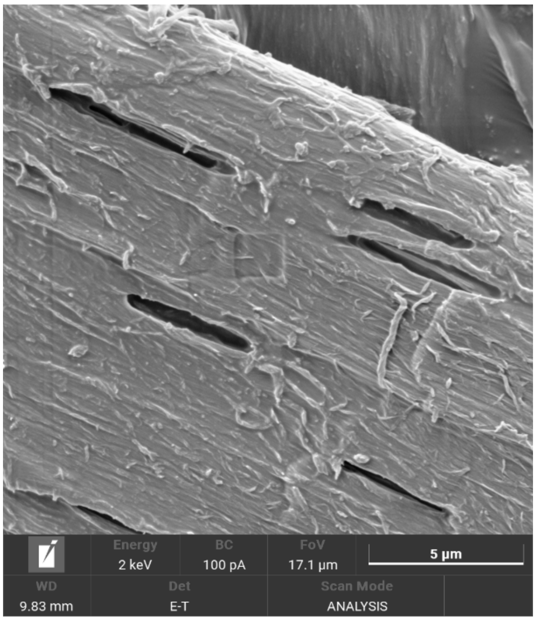

(c)

Figure S2. SEM images: (a) cellulose after 2-h hydrolysis; (b) cellulose after 8-h hydrolysis; and (c) cellulose after 24-h hydrolysis
